# Deep learning prediction of hospital readmissions for asthma and COPD

**DOI:** 10.1186/s12931-023-02628-7

**Published:** 2023-12-13

**Authors:** Kevin Lopez, Huan Li, Zachary Lipkin-Moore, Shannon Kay, Haseena Rajeevan, J. Lucian Davis, F. Perry Wilson, Carolyn L. Rochester, Jose L. Gomez

**Affiliations:** 1 Pulmonary, Critical Care and Sleep Medicine Section, Yale University, 300 Cedar Street, New Haven, CT 06520-8057 USA; 2 Center for Precision Pulmonary Medicine (P2MED), Yale University, New Haven, CT 06520 USA; 3 Cooley Dickinson Hospital, Northampton, MA 01060 USA; 4 Biomedical Informatics and Data Science, Yale University, New Haven, CT 06520 USA; 5 Epidemiology of Microbial Diseases, Yale School of Public Health, New Haven, CT 06520 USA; 6 Clinical and Translational Research Accelerator, Department of Medicine, Yale University, New Haven, CT 06520 USA; 7 VA Connecticut Healthcare System, West Haven, CT 06516 USA

## Abstract

**Question:**

Severe asthma and COPD exacerbations requiring hospitalization are linked to increased disease morbidity and healthcare costs. We sought to identify Electronic Health Record (EHR) features of severe asthma and COPD exacerbations and evaluate the performance of four machine learning (ML) and one deep learning (DL) model in predicting readmissions using EHR data.

**Study design and methods:**

Observational study between September 30, 2012, and December 31, 2017, of patients hospitalized with asthma and COPD exacerbations.

**Results:**

This study included 5,794 patients, 1,893 with asthma and 3,901 with COPD. Patients with asthma were predominantly female (n = 1288 [68%]), 35% were Black (n = 669), and 25% (n = 479) were Hispanic. Black (44 vs. 33%, p = 0.01) and Hispanic patients (30 vs. 24%, p = 0.02) were more likely to be readmitted for asthma. Similarly, patients with COPD readmissions included a large percentage of Blacks (18 vs. 10%, p < 0.01) and Hispanics (8 vs. 5%, p < 0.01). To identify patients at high risk of readmission index hospitalization data of a subset of 2,682 patients, 777 with asthma and 1,905 with COPD, was analyzed with four ML models, and one DL model. We found that multilayer perceptron, the DL method, had the best sensitivity and specificity compared to the four ML methods implemented in the same dataset.

**Interpretation:**

Multilayer perceptron, a deep learning method, had the best performance in predicting asthma and COPD readmissions, demonstrating that EHR and deep learning integration can improve high-risk patient detection.

**Supplementary Information:**

The online version contains supplementary material available at 10.1186/s12931-023-02628-7.

## Introduction

Asthma and chronic obstructive pulmonary disease (COPD) are the two most common chronic pulmonary diseases worldwide [1]. Health care expenses for asthma and COPD in 2020 were estimated to be $80 billion [2] and $49 billion in the United States alone [3]. Severe exacerbations that require hospitalization are linked to increased disease morbidity as well as increased healthcare cost [2, 3]. Rates of asthma exacerbation requiring emergency department visits or hospitalization range between 8.4% and 12.5% [4], and up to 20% for COPD [5]. Novel tools are therefore needed to improve disease management and facilitate therapeutic interventions.

Although asthma and COPD are both classified as obstructive lung diseases and share some clinical characteristics, their pathogenesis and therapies are vastly different. A major difference in COPD is the strong association with cigarette smoke exposure, which accounts for approximately 90% of cases in the US [6]. Efforts to improve asthma and COPD classifications have uncovered unique disease subtypes and endotypes. Endotypes are disease phenotypes characterized by similar biological mechanisms or responses to treatment [7]. This improvement in disease classification has enabled identification of individuals at risk for frequent exacerbations and comorbidities [8, 9]. These disease classification breakthroughs have also led to improved targeted therapeutics for both asthma and COPD [10–14]. Despite the importance of obstructive lung disease endotyping, systematic approaches that identify patients who are at high risk of recurrent adverse outcomes for both disorders are lacking. One of the reasons for the limited adoption of patient endotyping by physicians could be reproducibility issues [15].

Implementation of machine learning algorithms has been a key aspect of endotype identification in asthma and COPD [16–19]. However, these developments have been primarily confined to research studies and have not been translated into clinical practice. One way of addressing this translation gap is to use electronic health records (EHRs). The widespread use of EHRs allows high-throughput collection of clinical variables at distinct stages of healthcare delivery. Through EHR queries, computable phenotypes can be employed to identify clinical conditions [20]. These records are complex and difficult to analyze in large numbers by conventional approaches. However, machine and deep learning algorithms [21], can potentially use EHR analysis to support improved disease classification and clinical decision-making. Despite these potential benefits of EHR integration with machine and deep learning, understanding of the shared EHR-based features of severe asthma and COPD exacerbations is limited.

We hypothesized that patients with multiple hospitalizations for severe exacerbations of asthma and COPD, referred to as readmissions, would have distinct clinical characteristics that could be identified using a model trained on structured EHR data. To test this hypothesis, we applied machine and deep learning models to a cohort of patients hospitalized for asthma and COPD exacerbations. The resulting findings will allow the development of strategies that reduce severe disease exacerbations by establishing treatment pathways for patients with an increased risk of readmission. Improvements in disease care resulting from algorithmic development have the potential to lower disease morbidity and healthcare costs.

## Methods

### Data source and study population

We conducted a retrospective cohort study using data gathered from patients hospitalized at Yale-New Haven Hospital (YNHH) between September 30, 2012, after the Epic EHR system (Verona, WI) was implemented, and December 31, 2017. YNHH is a tertiary-care hospital with 1541 beds and two campuses in New Haven, Connecticut, USA. The Yale University Human Research Protection Program approved this study. Data was obtained from the Joint Data Analytics Team at Yale University School of Medicine. We included all participants who met the following criteria during the study period: This study was limited to hospital admissions of patients 12 years and over. The International Classification of Diseases, Tenth Revision, Clinical Modification (ICD-10-CM) codes were used for inclusion and exclusion, as indicated in Additional file 1: Table S1. Additional methods are presented in the Additional file 6.

For specific aspects of study design, we have included the Transparent Reporting of a multivariable prediction model for Individual Prognosis or Diagnosis (TRIPOD) checklist in the Additional file 2.

### Outcomes

Our primary outcome was the presence of more than one hospitalization for exacerbation of asthma or COPD, readmission, during the study period.

### Statistical analysis

Descriptive statistics used the Wilcoxon Rank Sum test for continuous values, chi-square for categorical values, two-proportions Z-test for proportions between groups. For each model, area under the curve (AUC) and confidence intervals (CI) for predicting patients with readmissions were calculated. Statistical significance was defined by p < 0.05. All statistical analyses were performed using R [22], version 3.6.3. We evaluated four machine learning algorithms including Naïve Bayes, support vector machine (SVM), random forest (RF), and gradient-boosted trees (GBT) and the deep learning model multilayer perceptron (MLP). We calculated SHapley Additive exPlanation (SHAP) values to interpret the deep learning MLP model [23]. SHAP values are measures of contributions each feature (predictor) has in the machine learning model. The rank order in every SHAP figure summarizes which feature values have the greatest influence on the prediction while accounting for the influence of all other feature values. The SHAP values show the distribution of each feature’s impact, and the color represents the feature value affecting the prediction (high = red; low = blue). The supplementary material includes a more detailed description of the methods.

## Results

### Demographic, comorbidities and hospitalization characteristics

This study included 5794 patients, 1893 with asthma, and 3901 with COPD. These patients accounted for a total of 10,464 hospitalizations during the study period. At the time of their index hospitalization, patients with asthma were younger than those with COPD (Table 1). Patients with asthma were predominantly female (n = 1288 [68%]), 35% were Black or African American (henceforth, Black) (n = 669), and 25% (n = 479) were Hispanic. Patients with COPD were also predominantly female (n = 2151 [55%]), however, unlike asthma patients, the majority were White (n = 3154 [81%]). There were significant differences in ever-smoking status between asthma (43%) and COPD (93%) (p < 0.01) (Table 1). COPD patients had higher rates of multiple comorbidities (n = 3467 [88%]) than asthma patients (n = 1133 [60%]) (p < 0.01) (Table 1).

**Table 1:** Demographics, comorbidities and medication administration

	Asthma	COPD	p-value
	N = 1893	N = 3901	
Age (years)	41 (26–58)	71 (61–81)	< 0.01
Female sex n (%)	1288 (68)	2151 (55)	< 0.01
Race n (%)			
African American/Black	669 (35)	500 (13)	< 0.01
White	770 (41)	3154 (81)	< 0.01
Other	403 (21)	220 (6)	< 0.01
Not Available*	53	27	
Hispanic Ethnicity n (%)	479 (25)	234 (6)	< 0.01
BMI (kg/m^2^)	30 (25–37)	27 (23–34)	< 0.01
Smoking status (n)	1812	3778	< 0.01
Never n (%)	1022 (56)	287 (7)	
Current n (%)	326 (18)	971 (26)	
Former n (%)	464 (25)	2520 (67)	
Length of Stay	2 (2–4)	4 (3–8)	< 0.01
ICU admission n (%)	267 (14)	880 (23)	< 0.01
Death during hospitalization n (%)	7 (0.4)	111 (3)	0.51
Death within a year n (%)	48 (3)	721 (18)	< 0.01
Readmission within 30 days n (%)	72 (4)	214 (6)	< 0.01
Multiple hospitalizations n (%)	386 (21)	1359 (35)	< 0.01
**Comorbidities**			
CAD	330 (17)	2026 (52)	< 0.01
CHF	270 (14)	2011 (52)	< 0.01
Cerebrovascular disease	200 (11)	770 (20)	< 0.01
Diabetes Mellitus	739 (39)	1960 (50)	< 0.01
CKD	238 (13)	1391 (36)	< 0.01
Allergic rhinitis	497 (26)	312 (8)	< 0.01
Nasal polyposis	34 (2)	31 (0.8)	< 0.01
Lung cancer	16 (0.8)	559 (14)	< 0.01
Sleep apnea	474 (25)	1322 (34)	< 0.01
GERD	836 (44)	2033 (52)	< 0.01
Hypertension	976 (52)	3399 (87)	< 0.01
Multiple comorbidities	1133 (60)	3467 (89)	< 0.01
**Medications during hospitalization**			
	N = 1856	N = 3863	
Albuterol	1481 (80)	2547 (66)	< 0.01
Theophylline	18 (1)	113 (3)	< 0.01
Antibiotic	993 (54)	3362 (87)	< 0.01
ICS	349 (19)	436 (11)	< 0.01
ICS + LABA	892 (48)	2498 (65)	< 0.01
LAMA	122 (7)	1758 (46)	< 0.01
LAMA + LABA	0	4 (0.1)	0.39
LTRA	572 (31)	512 (13)	< 0.01
Nicotine replacement	159 (9)	809 (21)	< 0.01
Systemic steroids	1433 (77)	3033 (79)	0.2527
Varenicline	0	1	1
Roflumilast	0	24	< 0.01

*Unknown or patient refused

*BMI* body mass index, *ICU* intensive care unit, *CAD* coronary artery disease, *CHF* congestive heart failure, *CKD* chronic kidney disease, *GERD* gastroesophageal reflux disease, *ICS* inhaled corticosteroid, *LABA* long-acting beta-agonist, *LAMA* long-acting muscarinic antagonist, *LTRA* leukotriene receptor antagonist

The median hospital length of stay for COPD exacerbations was longer than asthma exacerbations. Rates of admission to the intensive care unit (ICU), readmission within 30 days of discharge, and mortality during hospitalization were also higher for the COPD cohort (Table 1). There were significant differences in one-year mortality following index hospitalization between COPD (n = 721 [18%]) and asthma (n = 48 [3%]) (p < 0.01). Individuals with COPD (n = 1359 [35%]) had a higher percentage of 30-day readmissions than those with asthma (n = 386 [21%]) (p < 0.01) (Table 1).

### Inpatient medication use

Qualitative data on inpatient medication use was available for 1,856 (98%) asthma patients and 3,863 (99%) COPD patients. Patients with asthma received more inhaled corticosteroids (ICS). However, use of ICS combined with long-acting beta-agonist (LABA) (ICS/LABA) was higher in COPD. Despite these differences in inhaled therapy, systemic steroid administration during hospitalization for asthma and COPD was comparable. Antibiotic use was higher in COPD than in asthma (Table 1).

### Laboratory testing

To identify differences in blood leukocyte counts, we analyzed data from 777 patients with asthma and 1,905 patients with COPD, for whom results were available on the first day of the index hospitalization (Table 2). The overall white blood cell (WBC) counts did not differ between groups. Absolute neutrophil and monocyte counts, however, were higher in COPD, whereas absolute eosinophil, basophil, and lymphocyte counts were higher in asthma (Table 2). Rhinovirus was the most prevalent viral pathogen discovered in asthma and COPD exacerbations (Table 2).

**Table 2:** Laboratory values

	Asthma	COPD	p-value
Admission values	777	1905	
White blood cells	9600 [7300–12,500]	9900 [7400–12,900]	< 0.01
Absolute neutrophil count	6600 [4600–10,000]	7300 [5000–10,500]	< 0.01
Absolute eosinophil count	111 [0–291]	86 [0–211]	< 0.01
Absolute basophil count	20 [0–58]	8 [0–56]	< 0.01
Absolute monocyte count	616 [413–864]	726 [500–984]	< 0.01
Absolute lymphocyte count	1,489 [945–2309]	1,225 [790–1839]	< 0.01
Red blood cell count	4.5 [4.1–4.8]	4.2 [3.7–4.7]	< 0.01
Hematocrit	39 [36–42]	39 [34–43]	< 0.01
Hemoglobin	13.1 [11.8–14.1]	12.7 [11.1–14.2]	< 0.01
MCH	29.5 [27.9–30.8]	30.2 [28.5–31.8]	< 0.01
MCHC	33.2 [32.5–34.0]	32.9 [31.9–33.7]	< 0.01
MCV	88.6 [85.0–92.0]	92.0 [88.0–96.0]	< 0.01
Platelets	256 [209–314]	225 [172–290]	< 0.01
MPV	8.9 [7.7–10.2]	8.7 [7.6–9.9]	0.0531
**Viral testing**	N = 382	N = 561	
Viral positivity	125 (33)	128 (23)	< 0.01
Adenovirus	2 (0.5) 2% of + viral	0	
Influenza A	10 (3) 8% of +	18 (3) 14% of +	
Influenza B	3 (1) 2% of +	5 (1) 4% of +	
Influenza A/B	26 (7) 21% of +	20 (4) 16%	0.03
Metapneumovirus	4 (1) 3% of +	10 (2) 8%	
Parainfluenza	10 (3) 8% of +	22 (4) 17%	
Rhinovirus	48 (13) 38% of +	32 (6) 25%	< 0.01
RSV	23 (6) 18% of +	24 (4) 19%	

*MCH* mean corpuscular hemoglobin, *MCHC* mean corpuscular hemoglobin concentration, *MCV* mean corpuscular volume, *MPV* mean platelet volume, *RSV* respiratory syncytial virus

### Clinical features of patients with single versus multiple hospitalizations

To determine whether there were clinical differences between patients with single hospitalization for severe exacerbation of asthma and COPD, we compared their clinical characteristics (Table 3 and Additional file 1: Table S2). Patients with asthma readmissions were more likely to be Black or Hispanic than those with a single hospitalization. Patients with readmissions had increased rates of several comorbidities and increased use of disease-specific therapies (Table 3 and Additional file 1: Table S2). Patients who had readmissions for asthma had higher absolute eosinophil counts, absolute lymphocyte counts, and platelets, than those with single hospitalization. In contrast, patients with readmissions had a lower rate of viral positivity than those with a single hospitalization (Table 3 and Additional file 1: Table S2).

**Table 3:** Multiple admissions clinical characteristics

	Asthma			COPD		
	Single Hx	Multiple Hx	p-val	Single Hx	Multiple Hx	p-val
Number	1507	386		2542	1359	
Age (years)	41 (26–58)	41 (24–57)	0.47	72 (63–81)	70 (60–79)	< 0.01
Female sex n (%)	1023 (68)	265 (69)	0.82	1352 (53)	560 (41)	< 0.01
Race n (%)			< 0.01			< 0.01
African American	500 (33)	169 (44)		255 (10)	245 (18)	
White	652 (43)	117 (30)		2127 (84)	1027 (76)	
Hispanic ethnicity n (%)	363 (24)	116 (30)	0.02	127 (5)	107 (8)	< 0.01
BMI (kg/m^2^)	30.7 (25.3–37.2)	29.8 (25.1–36.7)	0.61	27.3 (22.6–33.2)	28.0 (23.2–34.3)	< 0.01
Smoking status (n)	1440	372	0.04	2435	1343	< 0.01
Never n (%)	806 (56)	216 (58)		214 (9)	73 (5)	
Current n (%)	275 (19)	51 (14)		633 (26)	338 (25)	
Former n (%)	359 (25)	105 (28)		1588 (65)	932 (69)	
Length of Stay	2 (2–4)	2 (2–4)	0.23	5 (3–8)	4 (3–7)	< 0.01
ICU admission n (%)	203 (13)	64 (17)	0.14	629 (25)	251 (18)	< 0.01
Death within a year n (%)	44 (3)	4 (1)	0.06	568 (22)	153 (11)	< 0.01
Multiple comorbidities (> 1)	870 (58)	263 (70)	< 0.01	2196 (86)	1271 (94)	< 0.01
Medications (n)	1475	381		2510	1353	
Albuterol	1112 (75)	370 (97)	< 0.01	1364 (54)	1183 (87)	< 0.01
ICS	226 (15)	123 (32)	< 0.01	193 (8)	243 (18)	< 0.01
ICS + LABA	604 (41)	288 (76)	< 0.01	1348 (54)	1150 (85)	< 0.01
LAMA	62 (4)	60 (16)	< 0.01	874 (35)	884 (65)	< 0.01
LTRA	353 (24)	220 (58)	< 0.01	206 (8)	306 (23)	< 0.01
Antibiotic	741 (50)	252 (66)	< 0.01	2041 (81)	1321 (98)	< 0.01
Nicotine replacement	107 (7)	52 (14)	< 0.01	405 (16)	4040 (30)	< 0.01
Systemic steroids	1073 (73)	361 (95)	< 0.01	1738 (69)	1295 (96)	< 0.01
CBC (n)	**627**	**150**		**1270**	**635**	
White blood cells	9.6 (7.4–12.7)	9.7 (7.1–12.1)	0.69	9.9 (7.3–13.0)	9.9 (7.6–12.8)	0.96
Absolute neutrophil count	6.7 (4.7–10.2)	6.2 (4.5–9.2)	0.13	7.4 (5.0–10.7)	7.2 (5.1–10.1)	0.22
Absolute eosinophil count	100 (0–273)	162 (0–367)	0.02	82 (0–201)	95 (0–233)	0.21
Absolute Lymphocyte count	1444 (924–2207)	1848 (1050–2498)	< 0.01	1173 (770–1785)	1728 (833–1985)	< 0.01
Hemoglobin	13.0 (11.8–14.1)	13.5 (12.3–14.3)	0.01	12.6 (11.0–14.3)	12.8 (11.3–14.1)	0.15
Platelets	252 (202–310)	272 (232–325)	< 0.01	225 (172–287)	226 (173–299)	0.31
Viral testing (n)	315	67		379	182	
Viral positivity	113 (36)*	12 (18)	0.02	98 (26)	30 (16)	0.03
Rhinovirus	46 (41)	2 (17)	0.02	27	5	0.06

*One instance of dual viral infection

*Hx* hospitalization, *MCH* mean corpuscular hemoglobin, *MCHC* mean corpuscular hemoglobin concentration, *MCV* mean corpuscular volume, *MPV* mean platelet volume

Patients with COPD readmissions had several similarities with patients readmitted for asthma, including a larger percentage of Black and Hispanic individuals. Furthermore, patients with COPD readmissions were younger and predominantly male compared to those with a single hospitalization. Patients with readmissions had a shorter length of stay and a lower rate of ICU admission. Patients with single hospitalizations had higher rates of mortality in the first year following index hospitalization compared to those with readmissions, likely reflecting the competition between mortality and readmissions (Table 3 and Additional file 1: Table S2). Patients with COPD readmissions had greater rates of multiple comorbidities and inpatient drug administration, similar to patients with asthma (Table 3 and Additional file 1: Table S2). Unlike asthma patients, patients with readmissions for COPD had absolute eosinophil counts comparable to those with a single hospitalization (95 vs. 82 cells/μL, p = 0.2).

### Predictive models to identify multiple exacerbators using index hospitalization information

**Fig. 1:**
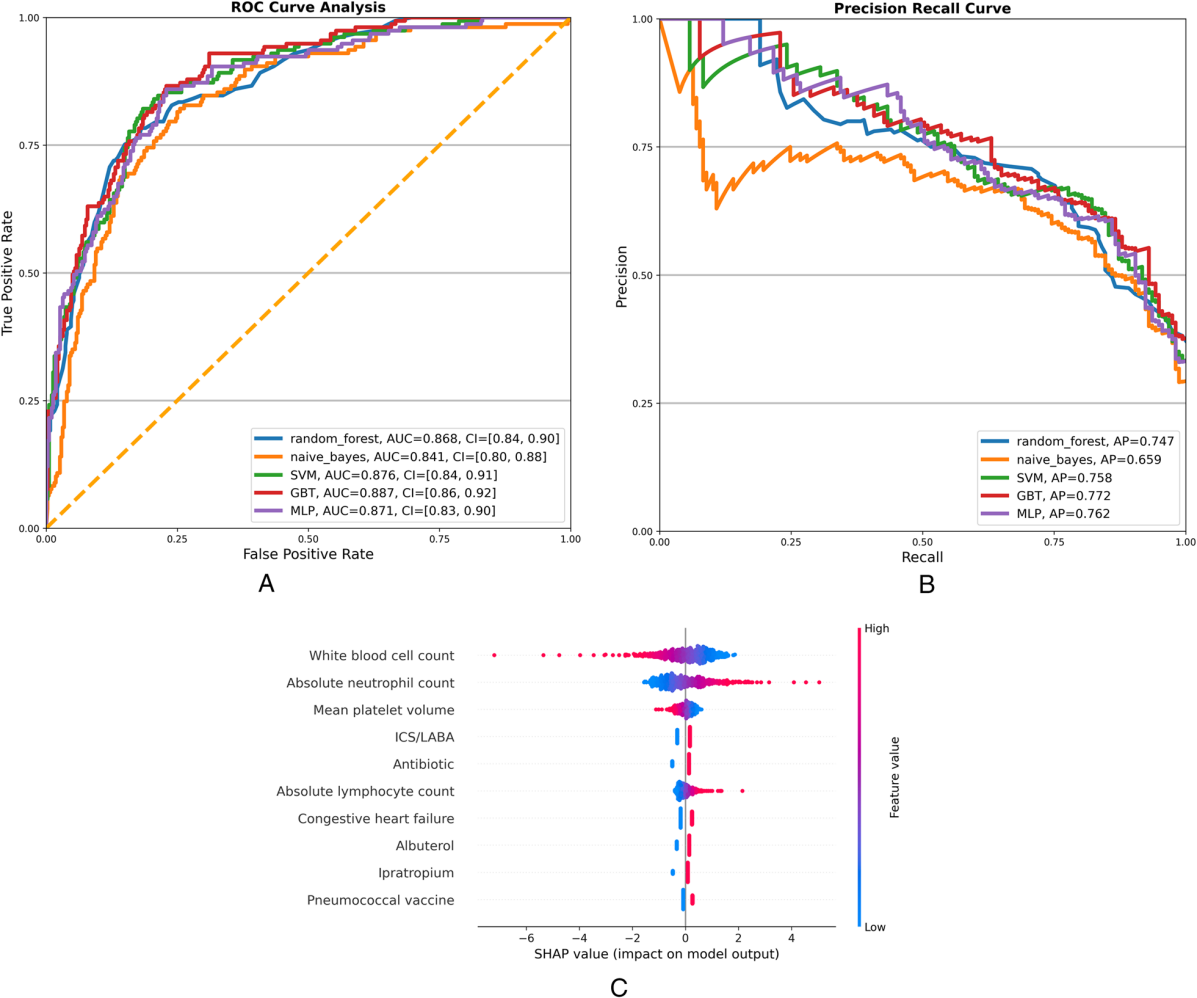
**A** Receiver operating characteristic (ROC) curves of four machine learning models and a deep learning model to predict readmissions in the combined cohort (n = 2682) of asthma (n = 777) and COPD (n = 1905). **B** Precision-recall (PR) curves of five machine learning models implemented in the combined cohort. **C** SHapley Additive exPlanation (SHAP) values of the top 10 predictive features of the multilayer perceptron (MLP) model implemented in the combined cohort

To identify patients at high risk for readmissions based on their index hospitalization data, we used machine learning (n = 4) and deep learning (n = 1) models. Our study examined a subset of asthma (n = 777) and COPD (n = 1905) patients with complete data on 60 variables (Additional file 1: Table S3). Focusing on readily available EHR variables or those that require minimal transformation. In this combined subgroup, 785 patients (29%) experienced the readmission outcome. The performance of the five models examined is summarized in Figs. 1A and B, and Table 4. With AUC values greater than 0.83, all models demonstrated good discriminating accuracy in classifying patients with readmissions vs. single hospitalization. Given the imbalanced nature of our dataset, we generated precision-recall (PR) curves that show similar average precision (AP) values (area under the PR curve) [24], except for Naive-Bayes (AP = 0.659). Despite the similar values in AUC and AP values between four out of the five models, the deep learning MLP model had the best balance between sensitivity (79%) and specificity (79%) to identify hospital readmission.

**Table 4:** Machine learning and neural network model performance to identify patients with multiple hospitalizations for severe exacerbations of asthma and COPD at YNHH

	AUC	CI	Sensitivity	Specificity	Precision	Accuracy
Full model						
Random forest	0.87	0.83–0.90	50%	93%	0.76	0.81
Naïve Bayes	0.84	0.80–0.88	46%	93%	0.73	0.79
SVM	0.88	0.84–0.91	55%	93%	0.76	0.82
Gradient boosted trees	0.89	0.86–0.92	63%	90%	0.72	0.82
Multilayer perceptron	0.87	0.83–0.90	79%	79%	0.61	0.79
Asthma						
Random forest	0.81	0.73–0.89	18%	100%	1.00	0.83
Naïve Bayes	0.88	0.81–0.94	62%	90%	0.64	0.84
SVM	0.79	0.70–0.88	3%	100%	1.00	0.79
Gradient boosted trees	0.80	0.71–0.89	29%	98%	0.83	0.84
Multilayer perceptron	0.83	0.75–0.91	71%	84%	0.55	0.81
COPD						
Random forest	0.87	0.83–0.91	58%	90%	0.73	0.79
Naïve Bayes	0.83	0.79–0.87	13%	97%	0.67	0.69
SVM	0.88	0.85–0.91	62%	88%	0.72	0.80
Gradient boosted trees	0.88	0.84–0.91	69%	85%	0.69	0.80
Multilayer perceptron	0.87	0.84–0.91	84%	78%	0.65	0.80

*AUC* area under the curve, *CI* confidence intervals, *SVM* support-vector machine

We then used SHAP values to identify feature relevance in the MLP model (Fig. 1C). The rank order in Fig. 1C summarizes the top 10 features with the highest value on the prediction of the readmission outcome. White blood cell counts and mean platelet volumes with high values contributed negatively to the prediction, while low values contributed positively. As for neutrophil and lymphocyte counts, the opposite holds true. A positive contribution to the prediction was made by hospital administration of ICS/LABA, antibiotics, albuterol, ipratropium, and pneumococcal vaccine, as well as congestive heart failure. However, an asthma or COPD diagnosis also affected the predictive model (Additional file 3: Figure S1). Therefore, we implemented all predictive models on each condition to determine whether the deep learning model had similar performance.

**Fig. 2:**
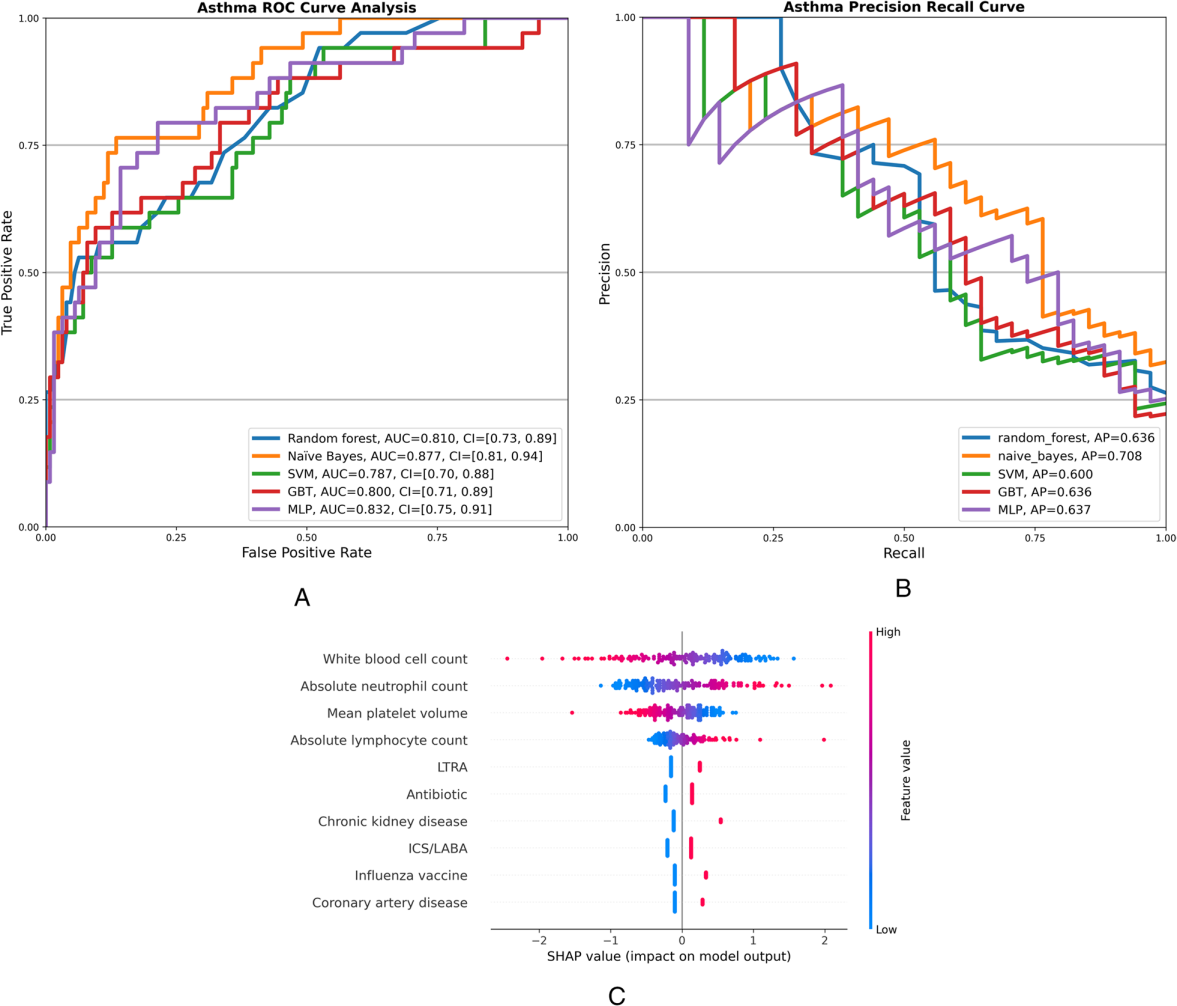
**A** ROC curves of four machine learning models and a deep learning model to predict readmissions in the asthma cohort (n = 777). **B** PR curves of five machine learning models implemented in the asthma cohort. C. SHAP values of the top 10 predictive features of the MLP model implemented in the asthma cohort

We constructed an asthma-only dataset (n = 777) and a COPD-only dataset (n = 1905) to assess the performance of the predictive models. In these datasets, 19% (n = 150) and 33% (n = 635) of patients suffered readmission, respectively. The performance values of each model are shown in Table 4. The AUCs were similar when the models were applied to the asthma cohort, Fig. 2A. While the naive Bayes model had a better AP, Fig. 2B, the MLP also had a more balanced performance, with a sensitivity of 71% and a specificity of 84%. SHAP values of the MLP model showed that CAD and CKD, as well as inpatient administration of LTRA and influenza vaccine, contributed positively to the prediction, and a subset of the top 10 features also had a similar directionality of effect on the asthma-specific prediction model as the full cohort model, Figs. 2C and Additional file 4: Figure S2).

**Fig. 3:**
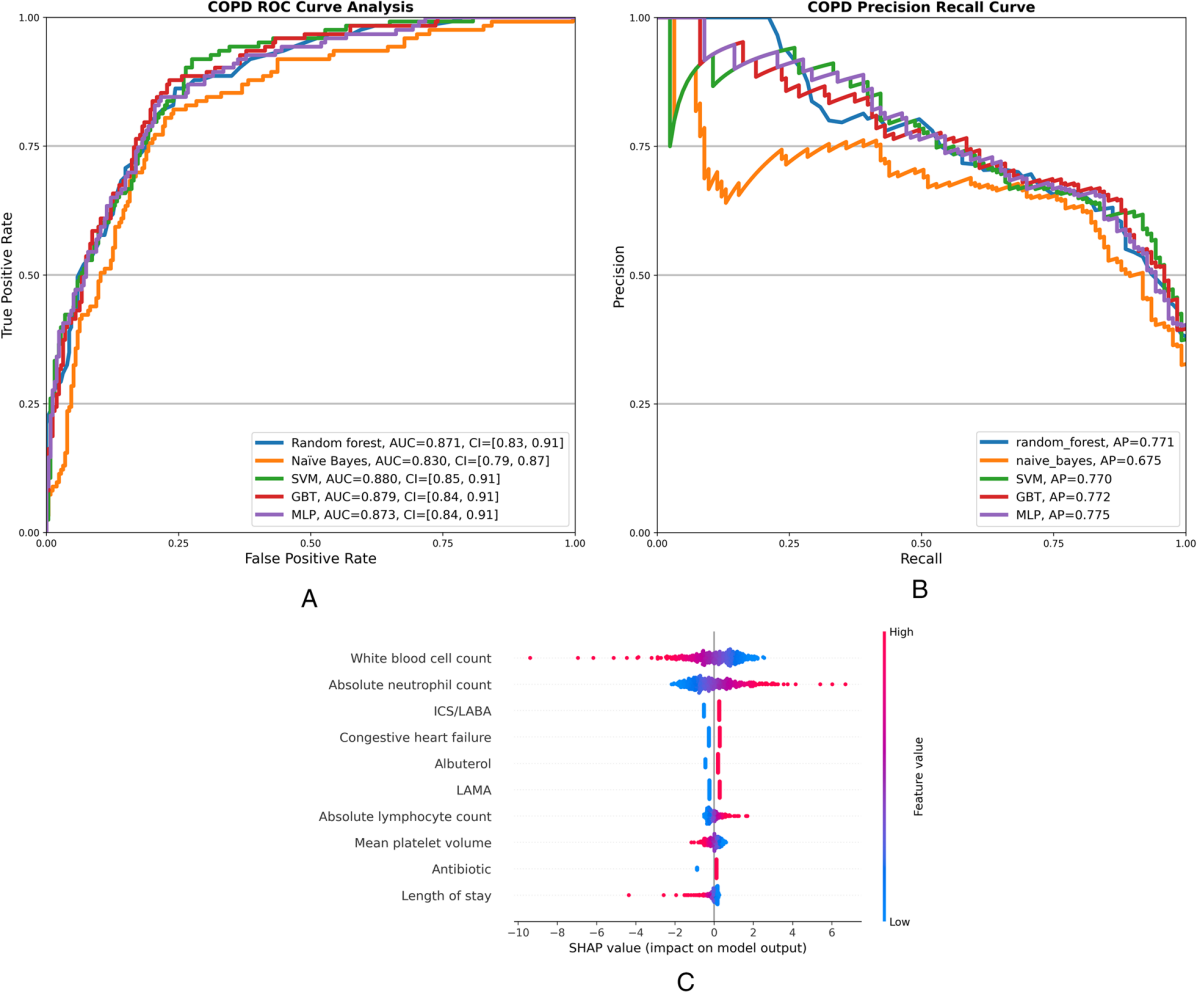
**A** ROC curves of four machine learning models and a deep learning model to predict readmissions in the COPD cohort (n = 1905). **B** PR curves of five machine learning models implemented in the COPD cohort. **C** SHAP values of the top 10 predictive features of the MLP model implemented in the COPD cohort

The models tested on the COPD-only dataset had similar AUC values over 0.83, Fig. 3A, but Naive Bayes’s AP value was significantly lower than all others at 0.675, Fig. 3B. Similarly to the full cohort and asthma datasets MLP had the best balance between sensitivity and specificity, 84% and 78% respectively, Table 4. A comparison of the top 10 SHAP features of the COPD MLP model with the full cohort showed the same effect of WBC, absolute neutrophils, and lymphocyte counts, mean platelet volume, and inpatient administration of ICS/LABA and albuterol, Fig. 3C and Additional file 5: Figure S3. Inpatient administration of LAMA and systemic steroids, however, significantly contributed to the readmission outcome. Longer hospital stays contributed negatively to the prediction, whereas shorter stays contributed positively. Together, these findings identify specific characteristics of index hospitalizations associated with risk of readmission that differ between asthma and COPD. Despite these unique features, a deep learning model incorporating both conditions is still capable of identifying patients at high risk for readmission with high sensitivity and specificity.

## Discussion

Our study found multiple common phenotypic features associated with readmissions among asthma and COPD patients. We explored various predictive models to identify patients at high risk of readmission. For these imbalanced datasets, Naive Bayes displayed the poorest performance among all models despite similar AUC metrics. Among all three datasets, MLP, a deep learning model, had the best balance between sensitivity and specificity. These results reveal that combining machine or deep learning models with computable EHR phenotypes and structured data from index asthma and COPD hospitalizations resulted in high predictive performance for identifying individuals at risk of readmission.

Due to the substantial morbidity and mortality associated with severe asthma and COPD exacerbations, identifying people at high risk is a top priority [2, 3]. Our models focused on the risk of readmissions. Our cohort’s phenotypic characteristics are similar to previous studies that have identified frequent exacerbator phenotypes in both asthma and COPD [8, 9]. During the index visit, multiple exacerbators had higher rates of congestive heart failure, inpatient administration of systemic steroids, antibiotics, LTRA, ICS, and ICS/LABA. Furthermore, individuals with multiple asthma and COPD exacerbations had higher absolute lymphocyte counts, which is a novel finding of unknown implications. Thus, individuals with asthma and COPD exacerbations share several clinical features associated with readmissions.

We also identified significant disparities in demographic characteristics among individuals with asthma and COPD exacerbations. Asthma exacerbations were common in women, accounting for two-thirds of all patients. A disproportionate number of Black and Hispanic patients were readmitted for asthma or COPD exacerbations, and non-Hispanic Blacks had a 43% rate of readmission compared to 26% in non-Hispanic whites (p < 0.01). These findings are consistent with prior studies [25, 26]. Closing these disparities should be a top priority for improving respiratory health equity. Although automated methods including those described here can help close disparities through automation, algorithms used in health systems are susceptible to biases that may affect high-risk groups disproportionately [27]. Algorithmic bias can be an unintended outcome of algorithmic development. As a result, when patient populations exhibit considerable disparities, such as in asthma and COPD readmissions, fairness-aware approaches to discover algorithmic bias [28, 29] should be used.

Given the widespread availability of EHRs and the potential to combine automated data collection with computable disease phenotypes and clinical care pathways, we sought to determine if this cohort of people with severe exacerbations could lead to better identification of patients who required readmission. Analytical tools such as machine learning and deep learning can maximize the use of big data in EHRs [30]. To imitate information received in real-time during hospitalization, we used minimum feature modification and structured data from EHRs. We also focused on data collected during a single index admission to identify patients at high risk of readmission. Differences in model performance may reflect the algorithms’ classification processes [31]. MLP, the deep learning model, had the best balance between sensitivity and specificity across several key metrics for classification compared with four machine learning algorithms. Among the machine learning algorithms, Naive Bayes had limitations in classifying subjects using the current data structure. A deep learning model with minimal transformation of structured EHR variables can identify individuals at high risk for asthma and COPD readmissions using their first hospitalization data with better performance than four commonly used machine learning algorithms.

SHAP analyses of MLP revealed specific features that recapitulate known frequent exacerbator phenotypes. Furthermore, data derived from complete blood counts was a strong feature in all models. These findings point to the potential presence of distinct immune and inflammatory profiles in individuals at high risk for readmission. These observations are complemented by previously described associations with specific comorbidities. Our findings have several implications. First, we found that our EHR-based study recapitulates multiple known features of frequent exacerbators in asthma and COPD. As a result, algorithms that quantitatively detect and analyze the range of features linked to a high risk of readmission can be implemented. Second, improved detection of high-risk individuals for readmissions can lead to personalized interventions to eliminate disparities. Finally, key features in our models can contribute to designing better predictive models and simplifying data collection. Existing and future deep learning advances integrated into EHRs have the potential to enhance clinical interactions in real time.

Our study has some limitations. First, while we used a stringent approach to identify asthma and COPD using a combination of ICD-10 codes and cigarette smoke exposure burden, the use of EHR data to establish disease groups may be associated with disease misclassification. However, our cohort’s patient characteristics are similar to those reported in past asthma and COPD studies. Second, there is a paucity of detailed information about outpatient therapy, as well as information on adherence to or using outpatient maintenance medications correctly. Third, we lack lung function data to assess the baseline illness severity in our population. Fourth, during their initial hospitalization, only a small number of individuals had viral testing performed. Fifth, we were unable to collect all readmissions for patients who were seen outside of our hospital network. However, because Yale-New Haven Health is our state’s largest healthcare system, the impact of this limitation is mitigated. Despite these limitations, we believe that implementing a standardized approach to patient identification, a common representation of data, and multiple model testing are strengths that balance these limitations. We are evaluating these results prospectively due to the evolving data representations and clinical practice changes.

### Interpretation

In this study of severe asthma and COPD exacerbations requiring hospitalization, we found that a deep learning algorithm had the best predictive performance over four machine learning models. These findings support the use of deep learning in conjunction with EHR adoption to prioritize care for individuals with a high risk of asthma and COPD readmission. The combination of deep learning with clinical decision support systems will result in the development of novel paradigms for treating asthma and COPD patients.

### Supplementary Information

**Additional file 1: **S1: ICD-10 codes for inclusion and exclusion criteria; S2: Clinical characteristics of multiple exacerbators; S3: Features and values used for the classifiers.

**Additional file 2: **Checklist based on Tripod guidelines for evaluation of models.

**Additional file 3: **SHapley Additive exPlanation (SHAP) values of all the predictive features of the multilayer perceptron (MLP) model implemented in the combined cohort.

**Additional file 4: **SHapley Additive exPlanation (SHAP) values of all the predictive features of the multilayer perceptron (MLP) model implemented in the asthma cohort.

**Additional file 5:** SHapley Additive exPlanation (SHAP) values of all the predictive features of the multilayer perceptron (MLP) model implemented in the COPD cohort.

**Additional file 6:** Supplementary methods.

## Data Availability

Access to data used for this publication has been limited to the PI and collaborators. Original approval from Yale University’s IRB did not include sharing provisions and is not applicable retroactively.

## Abbreviations

COPD: Chronic obstructive pulmonary disease
EHR: Electronic health record
ML: Machine learning
DL: Deep learning
ICD-10-CM: International classification of diseases, tenth revision, clinical modification
AUC: Area under the curve
CI: Confidence intervals
SVM: Support vector machine
RF: Random forest
GBT: Gradient-boosted trees
MLP: Multilayer perceptron
SHAP: SHapley additive explanation
WBC: White blood cell
PR: Precision-recall
AP: Average precision
CAD: Coronary artery disease
CKD: Chronic kidney disease
LTRA: Leukotriene receptor antagonists
ICS: Inhaled corticosteroid
LABA: Long-acting beta-agonist
LAMA: Long-acting muscarinic antagonist
