# Supplementary material for: Deep learning prediction of hospital readmissions for asthma and COPD
Source: Respir Res. 2023 Dec 13;24:311. doi: 10.1186/s12931-023-02628-7 (PMC10720134; doi:10.1186/s12931-023-02628-7)
Supplement: Supplementary file 2 — Additional file 2: Checklist based on Tripod guidelines for evaluation of models. [file 12931_2023_2628_MOESM2_ESM.docx]

| **Section/Topic** | **Item** |  | **Checklist Item** | **Page** |
| --- | --- | --- | --- | --- |
| **Title and abstract** | | | | |
| Title | 1 | D;V | Identify the study as developing and/or validating a multivariable prediction model, the target population, and the outcome to be predicted.  *Deep Learning Prediction of Hospital Readmissions for Asthma and COPD* | 1 |
| Abstract | 2 | D;V | Provide a summary of objectives, study design, setting, participants, sample size, predictors, outcome, statistical analysis, results, and conclusions.  *The abstract is being formatted per journal requirements and is provided in the manuscript.* | 2 |
| **Introduction** | | | | |
| Background and objectives | 3a | D;V | Explain the medical context (including whether diagnostic or prognostic) and rationale for developing or validating the multivariable prediction model, including references to existing models.  *The medical context for this paper is diagnostic to identify patients at high risk of hospital readmission for asthma and COPD exacerbations.*  *We have included specific examples in the introduction.* | 3-4 |
|  | 3b | D;V | Specify the objectives, including whether the study describes the development or validation of the model or both.  *Objectives: We proposed the hypothesis that patients with multiple hospitalizations for severe exacerbations of asthma and COPD would have distinct clinical characteristics and could be identified by machine learning model with EHR data.*  *In this study we are developing the machine learning model and learning which method is the best for this type of data and outcome.* | 3-4 |
| **Methods** | | | | |
| Source of data | 4a | D;V | Describe the study design or source of data (e.g., randomized trial, cohort, or registry data), separately for the development and validation data sets, if applicable.  *Study design is a retrospective cohort study*  *Data source is provided under the methods section* | 5-6, Supp. |
|  | 4b | D;V | Specify the key study dates, including start of accrual; end of accrual; and, if applicable, end of follow-up.  *The study date is between September 30, 2012 and December 31, 2017* | 5-6, Supp. |
| Participants | 5a | D;V | Specify key elements of the study setting (e.g., primary care, secondary care, general population) including number and location of centres.  *Hospital-based study. Two campuses located in New Haven, CT, USA.* | 5-6, Supp. |
|  | 5b | D;V | Describe eligibility criteria for participants.  *See Methods section.* | 5-6, Supp. |
|  | 5c | D;V | Give details of treatments received, if relevant.  *We collected information on treatments received while patients were hospitalized. We did not study any specific treatment intervention.* | 5-6, Supp. |
| Outcome | 6a | D;V | Clearly define the outcome that is predicted by the prediction model, including how and when assessed.  *Outcome: Identification of patients readmitted to the hospital during the study period.* | 5-6, Supp. |
|  | 6b | D;V | Report any actions to blind assessment of the outcome to be predicted.  *Analysts were not aware of the outcome while developing the model.* | Supp. |
| Predictors | 7a | D;V | Clearly define all predictors used in developing or validating the multivariable prediction model, including how and when they were measured.  *For specific details please refer to supplementary Table S3. These values were measured during patient’s first hospitalization.* | Supp. S3 |
|  | 7b | D;V | Report any actions to blind assessment of predictors for the outcome and other predictors.  *We did not select any specific predictors before developing the model and no linkage between predictors and outcomes was available during model development.* | Supp. |
| Sample size | 8 | D;V | Explain how the study size was arrived at.  *This was a secondary analysis of an existing cohort that is also described as part of this manuscript.* | 5 |
| Missing data | 9 | D;V | Describe how missing data were handled (e.g., complete-case analysis, single imputation, multiple imputation) with details of any imputation method.  *We only used complete data for these analyses and did not use imputation.* | 5 |
| Statistical analysis methods | 10a | D | Describe how predictors were handled in the analyses.  *For specific details please refer to supplementary Table E2. The predictor values were not transformed after extraction from the EHR.* | Supp. S3 |
|  | 10b | D | Specify type of model, all model-building procedures (including any predictor selection), and method for internal validation.  *Random Forest, Naïve Bayes, Support Vector Machines (SVM), Gradient Boosted Trees (GBT), and Multilayer Perceptron. We used a training and test sets that were derived from the same cohort.* | 5-6, Supp. |
|  | 10c | V | For validation, describe how the predictions were calculated. | 5-6, Supp. |
|  | 10d | D;V | Specify all measures used to assess model performance and, if relevant, to compare multiple models.  *We derived accuracy, sensitivity, specificity, area under the curve (AUC), and precision recall average precision (AP).* | 5-6, Supp. |
|  | 10e | V | Describe any model updating (e.g., recalibration) arising from the validation, if done.  *We did not recalibrate the model following validation. This is part of future work.* | N/A |
| Risk groups | 11 | D;V | Provide details on how risk groups were created, if done.  *N/A* | N/A |
| Development vs. validation | 12 | V | For validation, identify any differences from the development data in setting, eligibility criteria, outcome, and predictors.  *We used the same cohort for developing and validating the machine learning models (e.g., test and train).* | 5-6, Supp. |
| **Results** | | | | |
| Participants | 13a | D;V | Describe the flow of participants through the study, including the number of participants with and without the outcome and, if applicable, a summary of the follow-up time. A diagram may be helpful.  *Please see tables for specific details on the number of participants, frequency of the outcome. The follow-up was variable depending on when the first event was present during the study period.* | 7 and Tables 1, 2, 3 |
|  | 13b | D;V | Describe the characteristics of the participants (basic demographics, clinical features, available predictors), including the number of participants with missing data for predictors and outcome.  *Please see tables for specific details. We only used complete data for training and testing the model.* | Tables 1, 2, 3 |
|  | 13c | V | For validation, show a comparison with the development data of the distribution of important variables (demographics, predictors and outcome).  *We have a detailed description in results and tables.* | Tables 1, 2, 3 |
| Model development | 14a | D | Specify the number of participants and outcome events in each analysis.  *Asthma=777, outcome=150 (19%); COPD=1905, outcome=635 (33%).* | Tables 1, 2, 3 |
|  | 14b | D | If done, report the unadjusted association between each candidate predictor and outcome.  *N/A* | N/A |
| Model specification | 15a | D | Present the full prediction model to allow predictions for individuals (i.e., all regression coefficients, and model intercept or baseline survival at a given time point).  *We have reported the feature importance using SHAP models. These are included as figures in the main manuscript and supplement.* | Fig. 1 Supp. Figs |
|  | 15b | D | Explain how to the use the prediction model.  *The prediction model is based on structured EHR data. The model identifies patients at risk for readmission to the hospital using data collected during hospitalization for asthma and/or COPD exacerbations.* | 5-6, Supp. |
| Model performance | 16 | D;V | Report performance measures (with CIs) for the prediction model.  *Measures with Cis have been reported in the figures* | Fig. 1 |
| Model-updating | 17 | V | If done, report the results from any model updating (i.e., model specification, model performance).  *N/A* | N/A |
| **Discussion** | | | | |
| Limitations | 18 | D;V | Discuss any limitations of the study (such as nonrepresentative sample, few events per predictor, missing data).  *Limitations are presented in a specific paragraph of the discussion section.* | 13 |
| Interpretation | 19a | V | For validation, discuss the results with reference to performance in the development data, and any other validation data. | N/A |
|  | 19b | D;V | Give an overall interpretation of the results, considering objectives, limitations, results from similar studies, and other relevant evidence.  *We are presenting this in the discussion section.* | 11-13 |
| Implications | 20 | D;V | Discuss the potential clinical use of the model and implications for future research.  *At the end of the discussion section, we have suggested that there is a potential to enhance clinical decision by using real-time clinical decision support using the EHR and these novel machine learning models.* | 13 |
| **Other information** | | | | |
| Supplementary information | 21 | D;V | Provide information about the availability of supplementary resources, such as study protocol, Web calculator, and data sets.  *All the important figures and selected variables are provided in supplementary materials.* | Supp. |
| Funding | 22 | D;V | Give the source of funding and the role of the funders for the present study.  *R01 HL153604, and R03 HL154275 to JLG. P30 DK079310, R01 DK113191, and R01 HS027626 to FWP.* | 1 |

*Items relevant only to the development of a prediction model are denoted by D, items relating solely to a validation of a prediction model are denoted by V, and items relating to both are denoted D;V. We recommend using the TRIPOD Checklist in conjunction with the TRIPOD Explanation and Elaboration document.
