# Supplementary figures and images for: Deep learning prediction of hospital readmissions for asthma and COPD
Source: Respir Res. 2023 Dec 13;24:311. doi: 10.1186/s12931-023-02628-7 (PMC10720134; doi:10.1186/s12931-023-02628-7)

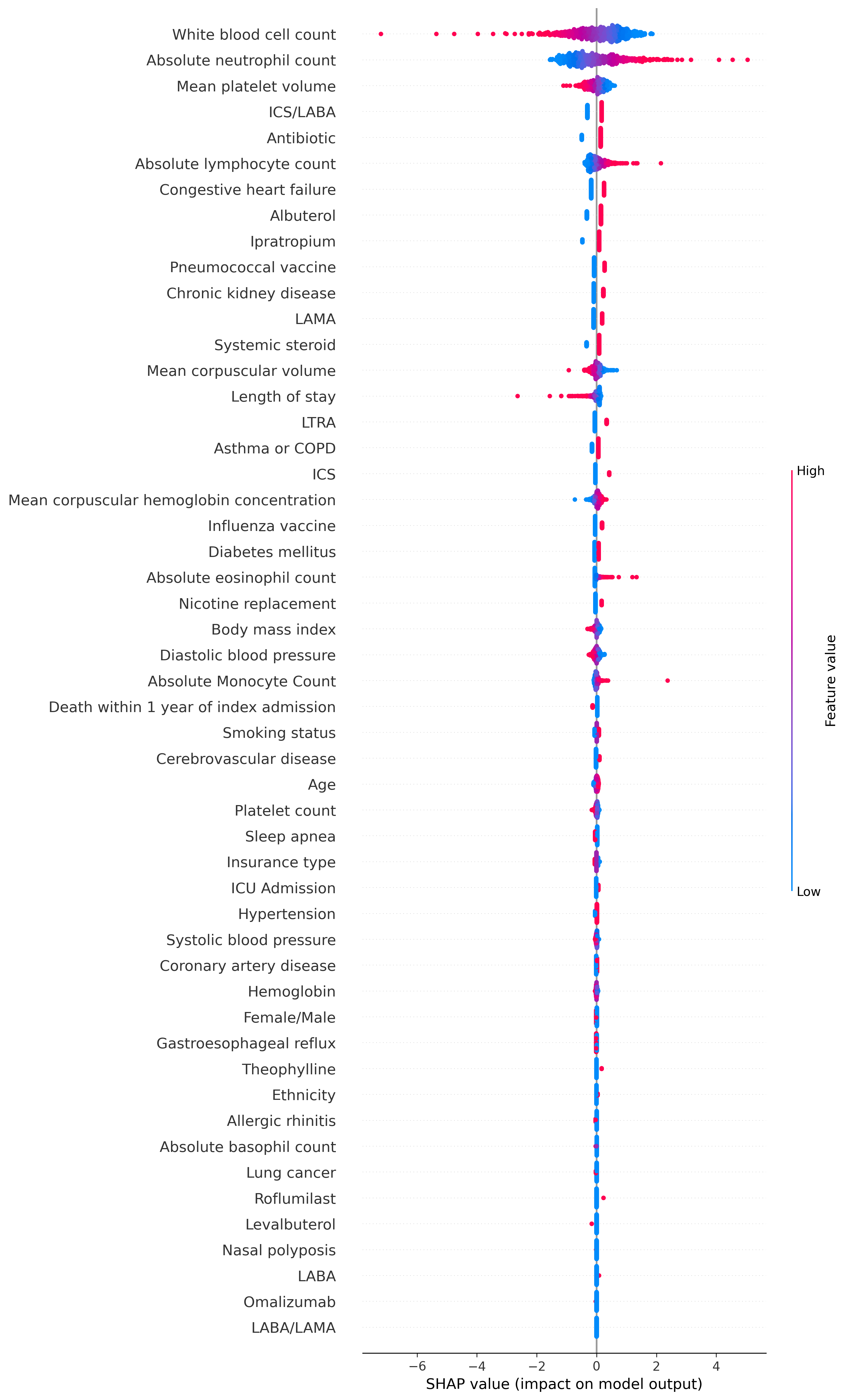

Supplement: Supplementary file 3 — Additional file 3: SHapley Additive exPlanation (SHAP) values of all the predictive features of the multilayer perceptron (MLP) model implemented in the combined cohort. [file 12931_2023_2628_MOESM3_ESM.png]

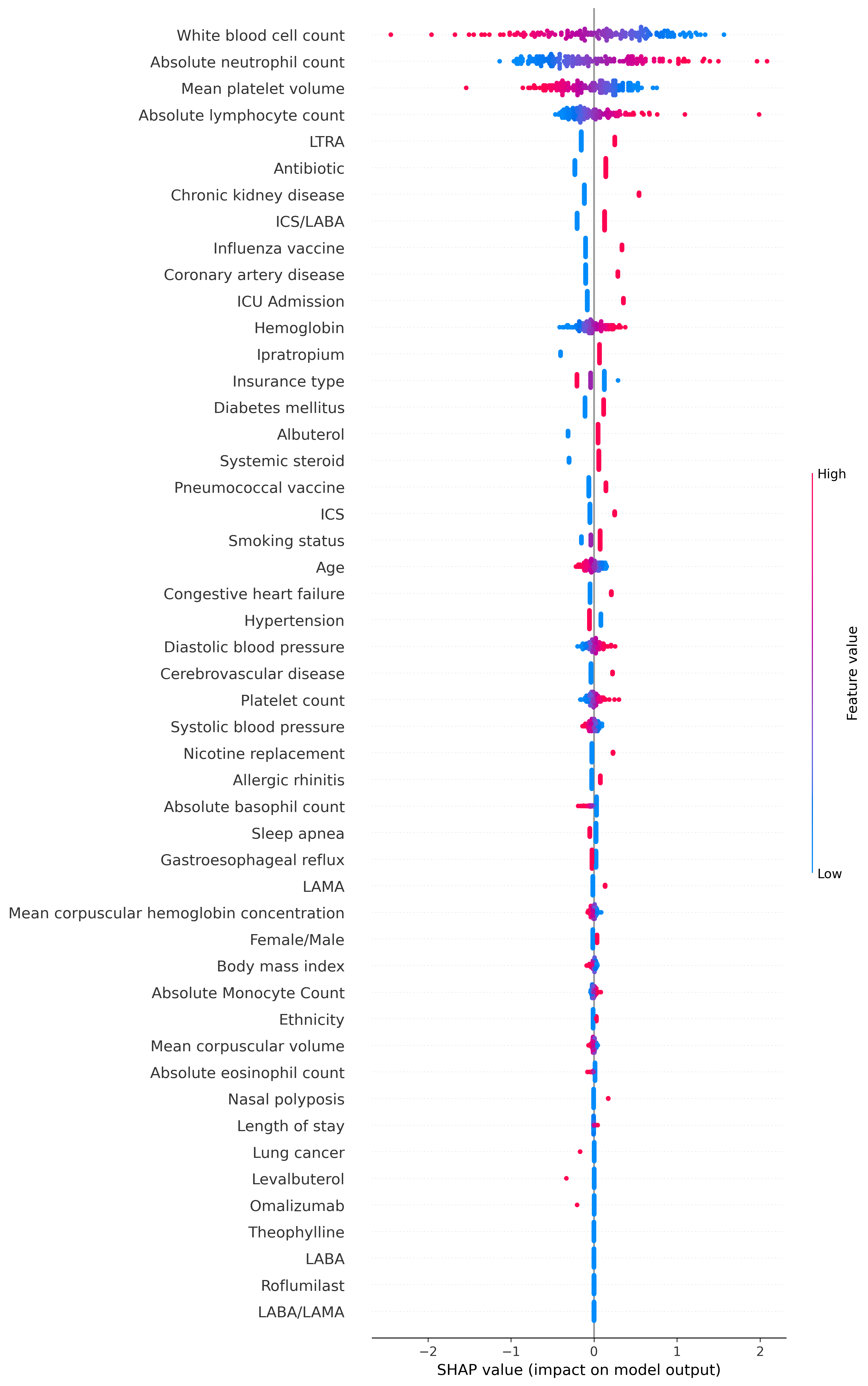

Supplement: Supplementary file 4 — Additional file 4: SHapley Additive exPlanation (SHAP) values of all the predictive features of the multilayer perceptron (MLP) model implemented in the asthma cohort. [file 12931_2023_2628_MOESM4_ESM.png]

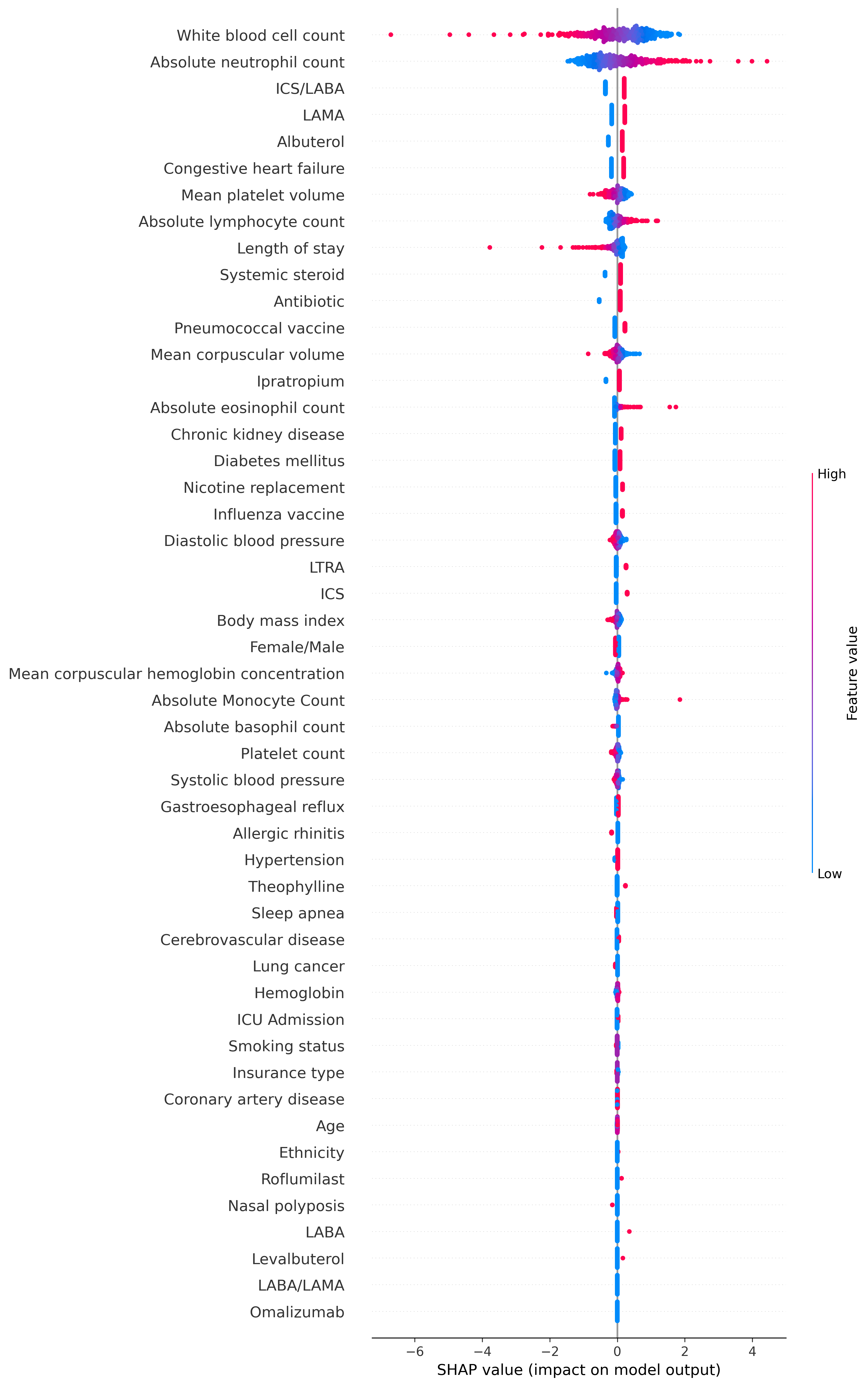

Supplement: Supplementary file 5 — Additional file 5: SHapley Additive exPlanation (SHAP) values of all the predictive features of the multilayer perceptron (MLP) model implemented in the COPD cohort. [file 12931_2023_2628_MOESM5_ESM.png]
