# Supplementary material for: Deep learning prediction of hospital readmissions for asthma and COPD
Source: Respir Res. 2023 Dec 13;24:311. doi: 10.1186/s12931-023-02628-7 (PMC10720134; doi:10.1186/s12931-023-02628-7)
Supplement: Supplementary file 6 — Additional file 6: Supplementary methods. [file 12931_2023_2628_MOESM6_ESM.docx]

**Data Source and Study Population**

The study was approved by the Yale Institutional Review Board under the title: ​​Analysis of asthma and chronic obstructive pulmonary disease exacerbations in EPIC at Yale New Haven Hospital, protocol ID 2000020165. Patients with asthma and a smoking history of more than 10 pack-years were excluded once the inclusion and exclusion criteria were determined. This procedure was intended to remove people who had asthma and COPD overlap syndrome or were misclassified as having asthma when they might instead have COPD. Patients with dual ICD-10-CM diagnosis for asthma and COPD at the time of the index or subsequent hospitalizations were also excluded from these analyses. The asthma cohort included patients with ICD-10-CM codes J45.901, J45.902, J45.21, J45.22, J45.32, J45.31, J45.42, J45.41, J45.51, J45.52 and a combination of unspecified asthma, uncomplicated (J45.909), and acute bronchitis (J20.90). The COPD cohort included patients with ICD-10-CM code J44.1 and patients with any combination of chronic obstructive pulmonary disease, unspecified (J44.9), unspecified chronic bronchitis (J42), emphysema, unspecified (J43.9), and acute bronchitis (J20.90). The first hospitalization that met inclusion and exclusion criteria during the study period was designated as the index hospitalization. Readmissions were defined as more than one hospitalization for asthma or COPD exacerbation during the study period. Following patient identification, demographic data, including self-reported race and self-reported Hispanic ethnicity, comorbidities, the first measured laboratory data, and inpatient medication records were collected from the index hospitalization. Comorbidity data were extracted from the EHR’s past medical history section. Laboratory data included complete blood count and viral testing. Viral testing used included antigen testing and PCR testing for common respiratory viral pathogens.

**Statistical Analysis**

The R packages dplyr v.0.8.5[[1]](https://paperpile.com/c/Htcs6M/VHtg8), lubridate v.1.7.9 [[2]](https://paperpile.com/c/Htcs6M/rv8PO) were used in this study. The python packages used in this study include Pandas v. 1.2.4, Numpy v. 1.20.2, Sklearn v. 0.24.2, Pytorch v. 1.9.0+cu102, torch_lr_finder v. 0.2.1, statsmodels v. 0.12.2, Matplotlib v. 3.4.1, pROC v 1.17.0.1, rpy2 v 3.4.5. The models trained here were run on Yale’s Milgram high performance computing cluster.

Given the presence of multiple shared features between patients with readmissions for asthma and COPD, we sought to evaluate whether machine learning and deep learning models would predict patients at risk for future readmissions using data from their first hospitalization for asthma or COPD exacerbation (index). Candidate predictors included 60 variables (**Supplementary Table 2**). For feature selection we excluded EHR data with Pearson correlation coefficients > 0.6, and clinically relevant variables. To avoid suboptimal representation of the outcome of interest from the training set in the test set, we split the dataset into stratified training (80%) and testing (20%), and we retained the same proportion of prediction targets in each. No individual patient could appear in both training and testing. Analysts were not aware of the outcome while developing the model. We did not select any specific predictors before developing the model and no linkage between predictors and outcomes was available during model development. We evaluated the performance of four machine learning (ML) algorithms and a deep learning algorithm for predicting the probability of readmissions during the study period. The machine learning algorithms included Naïve Bayes, support vector machine, random forest, and gradient-boosted trees. While the deep learning model was the multilayer perceptron (MLP) neural network. Despite the clinical differences between asthma and COPD, the combined models tested took into account each diagnosis, and were optimized to identify readmissions. Each model was trained using the train/test regime and tuned parameters. To make the best use of our data, we imputed missing data by replacing numeric variables with the mean and categorical variables with the mode **(Supplementary Table 2)**. Imputation used only training set values. We also set the random state of each machine to the same seed, specifically the number zero. The MLP model consisted of three layers: input, hidden, and output. In the MLP, we used the Elu activation function[[3]](https://paperpile.com/c/Htcs6M/CR888) and the Dropout regularization function with a probability of 0.3[[4]](https://paperpile.com/c/Htcs6M/BFKsj). For the loss function, we used CrossEntropyLoss and the ADAM optimizer[[5]](https://paperpile.com/c/Htcs6M/JKfKg) with a learning rate of 1e-3 and a weight decay of 1e-3. For the MLP, we experimented with different learning rates using the one cycle policy[[6]](https://paperpile.com/c/Htcs6M/4ED23). Given the class imbalance, we added weights to each class. On the training set, we used stratified 5-fold cross-validation to evaluate alternative parameters for our model. After validating our model, we trained the MLP and tested its performance on the withheld testing set.

**Brief machine learning and neural network description**

The random forest algorithm relies on decision trees, ensemble learning, a process in which multiple models are generated and trained on the same data, and bootstrapping. Random forest identifies the best classifier by randomly drawing decision trees and averaging them. Although gradient-boosted trees also use trees and ensemble learning, the trees are less complicated than those generated by random forest and are sometimes called stumps. Through iterative addition of these stumps, the model aims to reduce the loss function to minimize the classification error. Gradient-boosted trees are computationally intensive due to their iterative nature. Compared with gradient-boosted trees, Naïve Bayes is more efficient. Naïve Bayes is based on Bayes' Theorem of Independence, which assumes that all input features are independent and data properties independently contribute to the probability of a binary outcome. Considering the correlation between EHR features, the assumption of independence is violated in our dataset, limiting Naïve Bayes’ performance. In a support vector machine, all data points of one class are separated from those of the other class by a hyperplane in n-number of dimensions. This algorithm is memory efficient by using support vectors (a subset of data points closest to the hyperplane) in the decision function. The best hyperplane is the one that has the largest margin or distance between the two classes. Finally, MLP, the deep learning model, are feed forward neural networks in which all inputs are connected to hidden units (neural network layers) between inputs and outputs. In this neural network configuration, nonlinear activation functions are implemented in the hidden layers, with the output layer receiving and transforming the values from the last hidden layer.

**References**

1. [Hadley Wickham RF, Henry L, Müller K, Others. dplyr: A grammar of data manipulation. *Version 0. 7* 2018; 6.](http://paperpile.com/b/Htcs6M/VHtg8)

2. [Grolemund G, Wickham H, Others. Dates and times made easy with lubridate. *J. Stat. Softw.* 2011; 40: 1–25.](http://paperpile.com/b/Htcs6M/rv8PO)

3. [Clevert D-A, Unterthiner T, Hochreiter S. Fast and Accurate Deep Network Learning by Exponential Linear Units (ELUs) [Internet]. arXiv [cs.LG] 2015.Available from:](http://paperpile.com/b/Htcs6M/CR888) <http://arxiv.org/abs/1511.07289>[.](http://paperpile.com/b/Htcs6M/CR888)

4. [Srivastava N, Hinton G, Krizhevsky A, Sutskever I, Salakhutdinov R. Dropout: A Simple Way to Prevent Neural Networks from Overfitting. *J. Mach. Learn. Res.* 2014; 15: 1929–1958.](http://paperpile.com/b/Htcs6M/BFKsj)

5. [Kingma DP, Ba J. Adam: A Method for Stochastic Optimization [Internet]. arXiv [cs.LG] 2014.Available from:](http://paperpile.com/b/Htcs6M/JKfKg) <http://arxiv.org/abs/1412.6980>[.](http://paperpile.com/b/Htcs6M/JKfKg)

6. [Smith LN. Cyclical Learning Rates for Training Neural Networks [Internet]. arXiv [cs.CV] 2015.Available from:](http://paperpile.com/b/Htcs6M/4ED23) <http://arxiv.org/abs/1506.01186>[.](http://paperpile.com/b/Htcs6M/4ED23)
